# The role of machine learning in clinical research: transforming the future of evidence generation

**DOI:** 10.1186/s13063-021-05489-x

**Published:** 2021-08-16

**Authors:** E. Hope Weissler, Tristan Naumann, Tomas Andersson, Rajesh Ranganath, Olivier Elemento, Yuan Luo, Daniel F. Freitag, James Benoit, Michael C. Hughes, Faisal Khan, Paul Slater, Khader Shameer, Matthew Roe, Emmette Hutchison, Scott H. Kollins, Uli Broedl, Zhaoling Meng, Jennifer L. Wong, Lesley Curtis, Erich Huang, Marzyeh Ghassemi

**Affiliations:** 1 Duke Clinical Research Institute, Duke University School of Medicine, Box 2834, Durham, NC 27701 USA; 2 Department of Electrical Engineering and Computer Science, Massachusetts Institute of Technology, Cambridge, Massachusetts 02139 USA; 3 Institute for Medical Engineering and Science, Massachusetts Institute of Technology, Cambridge, Massachusetts 02139 USA; 4 CIFAR AI Chair, Vector Institute, Toronto Ontario, Canada; 5 Microsoft Research, Cambridge, MA USA; 6 AstraZeneca, Gothenburg, Sweden; 7 Courant Institute of Mathematical Science, New York University, New York, NY USA; 8 Englander Institute for Precision Medicine, Weill Cornell Medical College, New York, NY USA; 9 Northwestern University Clinical and Translational Sciences Institute, Northwestern University, Chicago, IL USA; 10 Division Pharmaceuticals, Open Innovation and Digital Technologies, Bayer AG, Wuppertal, Germany; 11 University of Alberta, Edmonton, Alberta Canada; 12 Department of Computer Science, Tufts University, Medford, MA USA; 13 Billion Minds, Inc., Seattle, WA USA; 14 Verana Health, San Francisco, CA USA; 15 Boehringer-Ingelheim, Burlington, Canada; 16 Sanofi, Cambridge, MA USA; 17 Sanofi, Washington, DC USA; 18 Duke Forge, Durham, NC USA; 19 Vector Institute, University of Toronto, Toronto, Ontario Canada

**Keywords:** Clinical trials as topic; Machine learning, Artificial intelligence, Research design, Research ethics

## Abstract

**Background:**

Interest in the application of machine learning (ML) to the design, conduct, and analysis of clinical trials has grown, but the evidence base for such applications has not been surveyed. This manuscript reviews the proceedings of a multi-stakeholder conference to discuss the current and future state of ML for clinical research. Key areas of clinical trial methodology in which ML holds particular promise and priority areas for further investigation are presented alongside a narrative review of evidence supporting the use of ML across the clinical trial spectrum.

**Results:**

Conference attendees included stakeholders, such as biomedical and ML researchers, representatives from the US Food and Drug Administration (FDA), artificial intelligence technology and data analytics companies, non-profit organizations, patient advocacy groups, and pharmaceutical companies. ML contributions to clinical research were highlighted in the pre-trial phase, cohort selection and participant management, and data collection and analysis. A particular focus was paid to the operational and philosophical barriers to ML in clinical research. Peer-reviewed evidence was noted to be lacking in several areas.

**Conclusions:**

ML holds great promise for improving the efficiency and quality of clinical research, but substantial barriers remain, the surmounting of which will require addressing significant gaps in evidence.

## Background

**Fig. 1:**
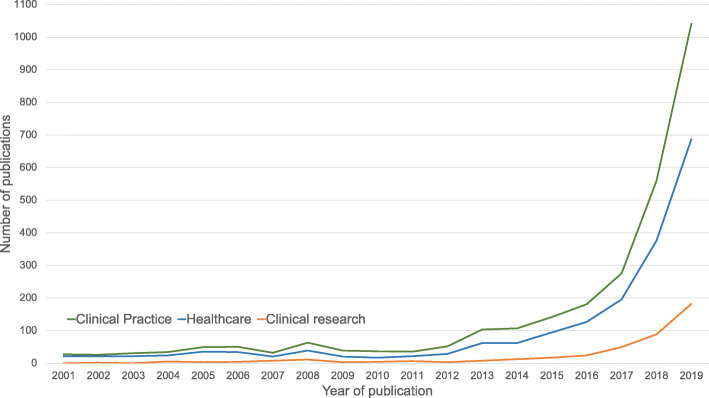
The number of clinical practice–related publications was determined by searching “(“machine learning” or “artificial intelligence”) and (“healthcare”).” The number of healthcare-related publications was determined by searching “(“machine learning” or “artificial intelligence”) and (“healthcare”)”, and the number of clinical research–related publications was determined by searching “(“machine learning” or “artificial intelligence”) and (“clinical research”).”

Interest in machine learning (ML) for healthcare has increased rapidly over the last 10 years. Though the academic discipline of ML has existed since the mid-twentieth century, improved computing resources, data availability, novel methods, and increasingly diverse technical talent have accelerated the application of ML to healthcare. Much of this attention has focused on applications of ML in healthcare *delivery*; however, applications of ML that facilitate clinical *research* are less frequently discussed in the academic and lay press (Fig. 1). Clinical research is a wide-ranging field, with preclinical investigation and observational analyses leading to traditional trials and trials with pragmatic elements, which in turn spur clinical registries and further implementation work. While indispensable to improving healthcare and outcomes, clinical research as currently conducted is complex, labor intensive, expensive, and may be prone to unexpected errors and biases that can, at times, threaten its successful application, implementation, and acceptance.

**Fig. 2:**
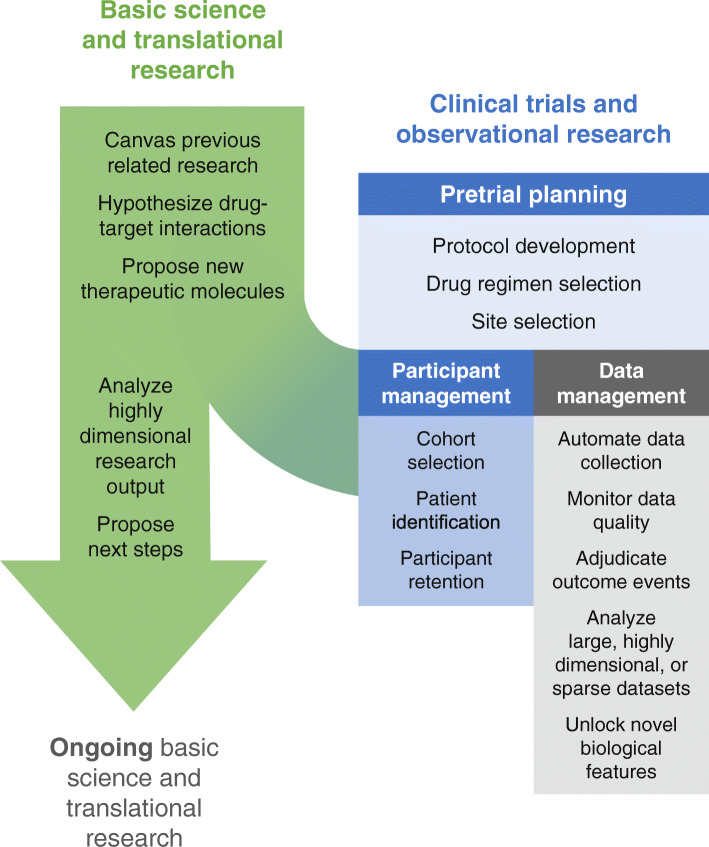
Areas of machine learning contribution to clinical research. Machine learning has the potential to contribute to clinical research through increasing the power and efficiency of pre-trial basic/translational research and enhancing the planning, conduct, and analysis of clinical trials

Machine learning has the potential to help improve the success, generalizability, patient-centeredness, and efficiency of clinical trials. Various ML approaches are available for managing large and heterogeneous sources of data, identifying intricate and occult patterns, and predicting complex outcomes. As a result, ML has value to add across the spectrum of clinical trials, from preclinical drug discovery to pre-trial planning through study execution to data management and analysis (Fig. 2). Despite the relative lack of academic and lay publications focused on ML-enabled clinical research (vìs-a-vìs the attention to ML in care delivery), the profusion of established and start-up companies devoting significant resources to the area indicates a high level of interest in, and burgeoning attempts to make use of, ML application to clinical research, and specifically clinical trials.

Key ML terms and principles may be found in Table 1. Many of the ML applications discussed in this article rely on deep neural networks, a subtype of ML in which interactions between multiple (sometimes many) hidden layers of the mathematical model enable complex, high-dimensional tasks, such as natural language processing, optical character recognition, and unsupervised learning. In January 2020, a diverse group of stakeholders, including leading biomedical and ML researchers, along with representatives from the US Food and Drug Administration (FDA), artificial intelligence technology and data analytics companies, non-profit organizations, patient advocacy groups, and pharmaceutical companies convened in Washington, DC, to discuss the role of ML in clinical research. In the setting of relatively scarce published data about ML application to clinical research, the attendees at this meeting offered significant personal, institutional, corporate, and regulatory experience pertaining to ML for clinical research. Attendees gave presentations in their areas of expertise, and effort was made to invite talks covering the entire spectrum of clinical research with presenters from multiple stakeholder groups for each topic. Subjects about which presentations were elicited in advance were intentionally broad and included current and planned applications of ML to clinical research, guidelines for the successful integration of ML into clinical research, and approaches to overcoming the barriers to implementation. Regular discussion periods generated additional areas of interest and concern and were moderated jointly by experts in ML, clinical research, and patient care. During the discussion periods, attendees focused on current issues in ML, including data biases, logistics of prospective validation, and the ethical issues associated with machines making decisions in a research context. This article provides a summary of the conference proceedings, outlining ways in which ML is currently being used for various clinical research applications in addition to possible future opportunities. It was generated through a collaborative writing process in which drafts were iterated through continued debate about unresolved issues from the conference itself. For many of the topics covered, no consensus about best practices was reached, and a diversity of opinions is conveyed in those instances. This article also serves as a call for collaboration between clinical researchers, ML experts, and other stakeholders from academia and industry in order to overcome the significant remaining barriers to its use, helping ML in clinical research to best serve *all* stakeholders.

**Table 1:** Key terms related to machine learning in clinical research

Term	Definition
Machine learning (ML)	A mathematical model that is able to improve its performance on a task by exposure to data.
Deep neural networks	ML models with one or more latent (hidden) layers allowing for the generation of non-linear output and complex interactions between layers. Deep neural networks power “deep learning,” which enables tasks, such as image recognition, natural language processing (NLP), and complex predictions. Subtypes of deep neural networks are classified based on the relationship between hidden layers and include convolutional, recurrent, gated graph, and generative adversarial neural networks.
Training, test, and validation sets	*Training set*: Dataset from which the model learns the optimal parameters to accomplish the task. *Test set*: Dataset on which the performance of a trained, parameterized model is evaluated. *Validation set*: Dataset that is used to evaluate the model’s performance during training. Differs from a test set in that it is used during training to establish hyperparameters of the model.
Supervised learning	A subset of ML in which the outcomes to be learned by the model (“labels”) are provided in the training set. For example, teaching a model to identify breast cancer patients for study inclusion would require training the model on a training set containing labeled patients with and without breast cancer prior to validating that model on a new set of *unlabeled* patients with and without breast cancer.
Unsupervised learning	A subset of ML in which there are no pre-specified labels for the model to learn to predict; instead, models identify hidden patterns in the data.
Natural language processing (NLP)	A form of artificial intelligence that enables the understanding of language. Much modern NLP uses deep neural networks in which words and their relationships to each other are encoded in a set of highly dimensional vectors, enabling the model to parse the meaning of new pieces of text it is presented with.

## The role of ML in preclinical drug discovery and development research

Successful clinical trials require significant preclinical investigation and planning, during which promising candidate molecules and targets are identified and the investigational strategy to achieve regulatory approval is defined. Missteps in this phase can delay the identification of promising drugs or doom clinical trials to eventual failure. ML can help researchers leverage previous and ongoing research to decrease the inefficiencies of the preclinical process.

### Drug target identification, candidate molecule generation, and mechanism elucidation

ML can streamline the process and increase the success of drug target identification and candidate molecule generation through synthesis of massive amounts of existing research, elucidation of drug mechanisms, and predictive modeling of protein structures and future drug target interactions [1]. Fauqueur et al. demonstrated the ability to identify specific types of gene-disease relationships from large databases even when relevant data-points were sparse [2], while Jia et al. were able to extract drug-gene-mutation interactions from the text of scientific manuscripts [3]. This work, along with other efforts to render extremely large amounts of biomedical data interpretable by humans [4, 5], helps researchers leverage and avoid duplicating prior work in order to target more promising avenues for further investigation. Once promising areas of investigation have been identified, ML also has a role to play in the generation of possible candidate molecules, for instance through use of a gated graph neural network to optimize molecules within the constraints of a target biological system [6]. In situations in which a drug candidate performs differently in vivo than expected, ML can synthesize and analyze enormous amounts of data to better elucidate the drug’s mechanism, as Madhukar et al. showed by applying a Bayesian ML approach to an anti-cancer compound [7]. This type of work helps increase the chance that drugs are tested in populations most likely to benefit from them. In the case of the drug evaluated by Madhukar et al., a better understanding of its mechanism facilitated new clinical trials in a cancer type (pheochromocytoma) more likely to respond to the drug (rather than prostate and endometrial cancers, among others).

Interpretation of large amounts of highly dimensional data generated during in vitro translational research (including benchtop biological, chemical, and biochemical investigation) informs the choice of certain next steps over others, but this process of interpretation and integration is complex and prone to bias and error. Aspuru-Guzik has led several successful efforts to use experimental output as input for autonomous ML-powered laboratories, integrating ML into the planning, interpretation, and synthesis phases of drug development [8, 9]. More recently, products of ML-enabled drug development have approached human testing. For example, an obsessive-compulsive personality disorder drug purportedly developed using AI-based methods is scheduled to begin phase I trials this year. The lay press reports that the drug was selected from among only 250 candidates and developed in only 12 months compared with the 2000+ candidates and nearly five years of development more typically required [10]. However, due to the lack of peer-reviewed publications about the development of this drug, the details of its development cannot be confirmed or leveraged for future work.

### Clinical study protocol optimization

As therapeutic compounds approach human trials, ML has a role to play in maximizing the success and efficiency of trials during the planning phase through application of simulation techniques to large amounts of data from prior trials in order to facilitate trial protocol development. For instance, study simulation may optimize the choice of treatment regimens for testing, as shown in a reinforcement learning approaches to Alzheimer’s disease and to non-small cell lung cancer [11, 12]. A start-up company called Trials.AI allows investigators to upload protocols and uses natural language processing to identify potential pitfalls and barriers to successful trial completion (such as inclusion/exclusion criteria or outcome measures) [13]. Unfortunately, performance of these example models has not been evaluated in a peer-reviewed manner, and they therefore offer only conceptual promise that ML in research planning can help ensure that a given trial design is optimally suited to the stakeholders’ needs.

In summary, there are clear opportunities to use ML to improve the efficiency and yield of preclinical investigation and clinical trial planning. However, most peer-reviewed reports of ML use in this capacity focus on preclinical research and development rather than clinical trial planning. This may be due to the greater availability of suitable large, highly dimensional datasets in translational settings in addition to greater potential costs, risks, and regulatory hurdles associated with ML use in clinical trial settings. Peer-reviewed evidence of ML application to clinical trial planning is needed in order to overcome these hurdles.

## The role of ML in clinical trial participant management

Clinical trial participant management includes the selection of target patient populations, patient recruiting, and participant retention. Unfortunately, despite significant resources generally being devoted to participant management, including time, planning, and trial coordinator effort, patient drop-out and non-adherence often cause studies to exceed allowable time or cost or fail to produce useable data. In fact, it has been estimated that between 33.6 and 52.4% of phase 1–3 clinical trials that support drug development fail to proceed to the next trial phase, leading to a 13.8% overall chance that a drug tested in phase I reaches approval [14]. ML approaches can facilitate more efficient and fair participant identification, recruitment, and retention.

### Selection of patient populations for investigation

Improved selection of specific patient populations for trials may decrease the sample size required to observe a significant effect. Put another way, improvements to patient population selection may decrease the number of patients exposed to interventions from which they are unlikely to derive benefit. This area remains challenging as prior work has discovered that for every 1 intended response, there are 3 to 24 non-responders for the top medications, resulting in a large number of patients who receive harmful side effects over the intended effect [15]. In addition to facilitating patient population selection through the rapid analysis of large databases of prior research (as discussed above), unsupervised ML of patient populations can identify patterns in patient features that can be used to select patient phenotypes that are most likely to benefit from the proposed drug or intervention [16]. Unstructured data is critical to phenotyping and identifying representative cohorts, indicating that considering additional data for patients is a crucial step toward identifying robust, representative cohorts [17]. For example, unsupervised learning of electronic health record (EHR) and genetic data from 11,210 patients elucidated three different subtypes of diabetes mellitus type II with distinct phenotypic expressions, each of which may have a different need for and response to a candidate therapy [18]. Bullfrog AI is a start-up that has sought to capitalize on the promise of targeted patient population selection, analyzing clinical trial data sets “to predict which patients will respond to a particular therapy in development, thereby improving inclusion/exclusion criteria and ensuring primary study outcomes are achieved” [19]. Though appealing in principle, this unsupported claim conflates outcome prediction (which is unlikely to succeed and runs counter to the intent of clinical research) with cohort selection (which would ideally identify patients on the basis of therapeutically relevant subtypes). Successfully identifying more selective patient populations does carry potential pitfalls: first, trials may be less likely to generate important negative data about subgroups that *would not* benefit from the intervention; and second, trials may miss subgroups who *would* have benefitted from the intervention, but whom the ML model missed. These potential pitfalls may be more likely to affect rural, remote, or underserved patient subgroups with more limited healthcare interactions. These two pitfalls carry possible implications for drug/device development regulatory approval and commercialization, as pivotal trials in more highly selected, and less representative, patient subgroups may require balancing the benefits of greater trial success with the drawbacks of more limited indications for drug/device use.

### Participant identification and recruitment

Once the specific cohort has been selected, natural language processing (NLP) has shown promise in identification of patients matching the desired phenotype, which is otherwise a labor-intensive process. For instance, a cross-modal inference learning model algorithm jointly encodes enrollment criteria (text) and patient records (tabular data) into a shared latent space, matching patients to trials using EHR data in a significantly more efficient manner than other machine learning approaches [20]. Some commercial entities offer similar services, including Mendel.AI and Deep6AI, though peer-reviewed evidence of their development and performance metrics is unavailable, raising questions about how these approaches perform [21, 22]. A potential opportunity of this approach is that it allows trialists to avoid relying on the completeness of structured data fields for participant identification, which has been shown to significantly bias trial cohorts [23, 24]. Unfortunately, to the extent that novel ML approaches to patient identification rely on EHRs, biases in the EHR data may affect the algorithms’ performances, leading to replacement of one source of bias (underlying the completeness of structured data) with another (underlying the generation of EHR documentation).

### Participant retention, monitoring, and protocol adherence

Two broad approaches are available to improve participant retention and protocol adherence using ML-assisted methods. The first is to use ML to collect and analyze large amounts of data to identify and intervene upon participants at high risk of study non-compliance. The second approach is to use ML to decrease participant study burden and thereby improve participants’ experiences.

AiCure is a commercial entity focused on protocol adherence using facial recognition technology to ensure patients take the assigned medication. AiCure was demonstrated to be more effective than a modified directly observed therapy strategy at detecting and improving patient adherence in both a schizophrenia trial and an anticoagulation trial among patients with a history of recent stroke [25, 26]. Unfortunately, AiCure’s model development and validation process has not been published, heightening concerns that it may perform differently in different patient subgroups, as has been demonstrated in other areas of computer vision [27]. Furthermore, these approaches, though promising, may encounter a potential barrier to implementation because their perceived invasiveness of privacy may not be acceptable to all research participants and because selecting patients with access to and comfort with the necessary devices and technology may introduce bias.

The other approach to improving participant retention uses ML to reduce the trial burden for participants using passive data collection techniques (methods will be discussed further in the “Data collection and management” section) and by extracting more information from available data generated during clinical practice and/or by study activities. Information created during routine clinical care can be processed using ML methods to yield data for investigational purposes. For instance, generative adversarial network modeling of slides stained with hematoxylin and eosin in the standard clinical fashion can detect which patients require more intensive and expensive multiplexed imaging, rather than subjecting all participants to that added burden [28]. NLP can also facilitate repurposing of clinical documentation for study use, such as auto-populating study case report forms, often through reliance on the Unified Medical Language System [29, 30]. Patients also create valuable content outside of the clinical trial context that ML can process into study data to reduce the burden of data collection for trial participants, such as natural language processing of social media posts to identify serious drug reactions with high fidelity [31]. Patient data from wearable devices have proven to be able to correlate participant activity with the International Parkinson and Movement Disorders Society Unified Parkinson’s Disease Rating Scale, distinguish between neuropsychiatric symptomatology patterns, and identify patient falls [32–34].

In summary, although ML and NLP have shown promise across a broad range of activities related to improving the management of participants in clinical trials, the implications of these applications of ML/NLP in regard to clinical trial quality and participant experience are unclear. Studies comparing different approaches to participant management are a necessary next step toward identifying best practices.

## Data collection and management

The use of ML in clinical trials can change the data collection, management, and analysis techniques required. However, ML methods can help address some of the difficulties associated with missing data and collecting real-world data.

### Collection, processing, and management of data from wearable and other smart devices

Patient-generated health data from wearable and other mobile/electronic devices can supplement or even replace study visits and their associated traditional data collection in certain situations. Wearables and other devices may enable the validation and use of new, patient-centered biomarkers. Developing new “digital biomarkers” from the data collected by a mobile device’s various sensors (such as cameras, audio recorders, accelerometers, and photoplethysmograms) often requires ML processing to derive actionable insights because the data yielded from these devices can be sparse as well as variable in quality, availability, and synchronicity. Using the relatively large and complex data yielded by wearables and other devices for research purposes therefore requires specialized data collection, storage, validation, and analysis techniques [34–37]. For instance, a deep neural network was used to process input from a mobile single-lead electrocardiogram platform [38], a random forest model was used to process audio output from patients with Parkinson’s disease [39], and a recurrent neural network was used to process accelerometer data from patients with atopic dermatitis [40]. These novel digital biomarkers may facilitate the efficient conduct and patient-centeredness of clinical trials, but this approach carries potential pitfalls. As has been shown to occur with an electrocardiogram classification model, ML processing of wearable sensor output to derive research endpoints introduces the possibility of corrupt results if the ML model is subverted by intentionally or unintentionally modified sensor data (though this risk exists with any data regardless of processing technique) [41]. Because of the complexity involved, software intended to diagnose, monitor, or treat medical conditions is regulated by the FDA, and the FDA has processes and guidance related to biomarker validation and qualification for use in regulatory trials.

Beyond the development of novel digital biomarkers, other device-related opportunities in patient centricity include the ability to export data and analytics back to participants to facilitate education and insight. Barriers to implementation of ML processing of device data include better defining how previously validated clinical endpoints and patient-centric digital biomarkers overlap as well as understanding participant opinions about privacy in relation to the sharing and use of device data. FDA approval of novel biomarkers will also be required. Researchers interested in leveraging the power of these devices must explain to patients their risks and benefits both for ethical and privacy-related reasons and because implementation without addressing participant concerns has the potential to *worsen* participant recruitment and retention [42].

### Study data collection, verification, and surveillance

An appealing application of ML, specifically NLP, to study data management is to automate data collection into case report forms, decreasing the time, expense, and potential for error associated with human data extraction, whether in prospective trials or retrospective reviews. Though this use requires overcoming variable data structures and provenances, it has shown early promise in cancer [43, 44], epilepsy [30], and depression [45], among other areas [29]. Regardless of how data have been collected, ML can power risk-based monitoring approaches to clinical trial surveillance, enabling the prevention and/or early detection of site failure, fraud, and data inconsistencies or incompleteness that may delay database lock and subsequent analysis. For instance, even when humans collect data into case report forms (often transmitted in PDF form), the adequacy of the collected data for outcome ascertainment can be assessed by combining optical character recognition with NLP [46]. Suspicious data patterns in clinical trials, or incorrect data in observational studies, can be identified by applying auto-encoders to distinguish plausible from implausible data [47].

### Endpoint identification, adjudication, and detection of safety signals

ML can also be applied to data processing. Semi-automated endpoint identification and adjudication offers the potential to reduce time, cost, and complexity compared with the current approach of manual adjudication of events by a committee of clinicians, because while endpoint adjudication has traditionally been a labor-intensive process, sorting and classifying events lies well within the capabilities of ML. For instance, IQVIA Inc. has described the ability to automatically process some adverse events related to drug therapies using a combination of optical character recognition and NLP, though this technique has not been described in peer-reviewed publications [48]. A potential barrier to implementation of semi-automated event adjudication is that endpoint definitions and the data required to support them often change from trial to trial, which theoretically requires re-training a classification model for each new trial (which is not a viable approach). More recently, efforts have been made to standardize outcomes in the field of cardiovascular research, though not all trials adhere to these outcomes. Trial data have not been pooled to facilitate model training for cardiovascular endpoints, and most fields have not yet undertaken similar efforts [49]. Further efforts in this area will require true consensus about event definitions, use of consensus definitions, and a willingness of stakeholders to share adequate data for model training from across multiple trials.

### Approaches to missing data

ML can be used in several different ways to address the problem of missing data, across multiple causes for data missingness, data-related assumptions and goals, and data collection and intended analytic methods. Possible goals may be to impute specific estimates of the missing covariate values directly or to average over many possible values from some learned distribution to compute other quantities of interest. While the latest methods are evolving and more systematic comparisons are needed, some early evidence suggests more complex ML methods may not always be of benefit over simpler imputation methods, such as population mean imputation [50]. Applications of missing value techniques include analysis of sparse datasets, such as registries, EHR data, ergonomic data, and data from wearable devices [51–54]. Although these techniques can help mitigate the negative effects of data missingness or scarcity, over-reliance on data augmentation methods may lead to the development of models with limited applicability to new, imperfect datasets. Therefore, a more meaningful approach would be to apply ML to improve data collection during the conduct of research itself.

### Data analysis

Data collected in clinical trials, registries, and clinical practices are fertile sources for hypothesis generation, risk modeling, and counterfactual simulation, and ML is well suited for these efforts. For instance, unsupervised learning can identify phenotypic clusters in real-world data that can be further explored in clinical trials [55, 56]. Furthermore, ML can potentially improve the ubiquitous practice of secondary trial analyses by more powerfully identifying treatment heterogeneity while still providing some protection (although incomplete) against false-positive discoveries, uncovering more promising avenues for future study [57, 58]. Additionally, ML is effectively used to generate risk predictions in retrospective datasets that can subsequently be prospectively validated. For instance, using a random forest model in COMPANION trial data, researchers were able to improve discrimination between patients who would do better or worse following cardiac resynchronization therapy compared with a multivariable logistic regression [59]. This demonstrates the ability of random forests to model interactions between features that are not captured by simpler models.

While predictive modeling is an important and necessary task, the derivation of real-world evidence from real-world data (i.e., making causal inferences) remains a highly sought-after (and very difficult) goal toward which ML offers some promise. Proposed techniques include optimal discriminant analysis, targeted maximum likelihood estimation, and ML-powered propensity score weighting [60–64]. A particularly intriguing technique involves use of ML to enable counterfactual policy estimation, in which existing data can be used to make predictions about outcomes under circumstances that do not yet, or could not, exist [65]. For instance, trees of predictors can offer survival estimates for heart failure patients under the conditions of receiving or not receiving a heart transplant and reinforcement learning suggests improved treatment policies on the basis of prior sub-optimal treatments and outcomes [66, 67]. Unfortunately, major barriers to implementation are a lack of interoperability between EHR data structures and fraught data sharing agreements that limit the amount of data available for model training [68].

In summary, there are many effective ML approaches to clinical trial data management, processing, and analysis but fewer techniques for improving the quality of data as they are generated and collected. As data availability and quality are the foundations of ML approaches, the conduct of high-quality trials remains of utmost importance to enable higher-level ML processing.

## Barriers to the integration of ML techniques in clinical research

Both operational and philosophical barriers limit the harnessing of the full potential of ML for clinical research. ML in clinical research is a high-risk proposition due to the potential to propagate errors or biases through multiple research contexts and into the corpus of biomedical evidence due to the use of flawed models; however, as previously discussed, ML offers promising ways to improve the quality and efficiency of clinical research for patients and other stakeholders. Both the operational and philosophical barriers to ML integration require attention at each stage of model development and use to overcome hurdles while maximizing stakeholder confidence in the process and its results. Operational barriers to ML integration in clinical research can aggravate and reinforce philosophical concerns if not managed in a robust and transparent manner. For instance, inadequate training data and poor model calibration can lead to racial bias in model application, such as has been noted in ML for melanoma identification [27]. Stakeholders, including regulatory agencies, funding sources, researchers, participants, and industry partners, must collaborate to fully integrate ML into clinical research. The wider ML community espouses “FAT (fairness, accountability, and transparency) ML” principles that also include responsibility, explainability, accuracy, auditability, and fairness and that should be applied to ML in clinical research, as discussed further.

### Operational barriers to ML in clinical research

The development of ML algorithms and their deployment for clinical research use is a multi-stage, multi-disciplinary process. The first step is to assemble a team with the clinical and ML domain expertise necessary for success. Failing to assemble such a team and to communicate openly within the team increases the risks of either developing a model that distorts clinical reality or using an ML technique that is inappropriate to the available data and research question at hand [69]. For instance, a model to predict mortality created without any clinical team members may identify intubation as predictive of mortality, which is certainly true but likely clinically useless. Collaboration is necessary and valuable for both the data science and clinical science components of the team but may require additional up-front, cross-disciplinary training, transparency, and trust to fully operationalize.

The choice and availability of data for algorithm development and validation is both a stubborn and highly significant barrier to ML integration into clinical research, though its full discussion is outside the scope of this manuscript. Many recent ML models, especially deep neural networks, require large amounts of data to train and validate. To ensure generalizability beyond the training data set, developers should use multiple data sources during this process because a number of documented cases demonstrated that algorithms performed significantly differently in validation data sets compared with training data sets [70]. Because data used in clinical research are often patient related and generated by institutions (in the case of EHR data) or companies (in the case of clinical trial data) at a significant cost, owners of data may be reluctant to share. Even when they are willing to share data, variation in data collection and storage techniques can hamper interoperability. Large datasets, such as MIMIC, eICU, and the UK Biobank, are good resources when other real-world data cannot be obtained [71–73], but any single data source is inadequate to yield a model that is ready for use, especially because training on retrospective data (such as MIMIC and UK Biobank) does not always translate well to prospective applications. For example, Nestor et al. demonstrated the importance of considering year of care in MIMIC due to temporal drift, and Gong et al. demonstrated methods for feature aggregation across large temporal changes, such as EHR transitions [70, 74]. Furthermore, certain disease states and patient types are less likely to be well represented in data generated for the purpose of clinical care. For example, while MIMIC is widely used because of its public availability, models trained on its ICU population are unlikely to generalize to many applications outside critical care. These issues with data availability and quality are intimately associated with problems surrounding reproducibility and replicability [75], which are more difficult to achieve in ML-driven clinical research for a number of reasons in addition to data availability, including the role of randomness in many ML techniques and the computational expense of model replication. The ongoing difficulties with reproducibility and replicability of ML-driven clinical research threaten to undermine stakeholder confidence in ML integration into clinical research.

### Philosophical barriers to ML in clinical research

*Explainability* refers to the concept that the processes underlying algorithmic output should be explainable to algorithm users in terms they understand. A large amount of research has been devoted to techniques to accomplish this, including attention scores and saliency maps, but concerns about the performance and suitability of these techniques persist [76–79]. Though an appealing principle, a significant debate exists about whether the concept of explainability interferes unnecessarily with the ability of ML to positively contribute to clinical care and research. Explainability may lead researchers to incorrectly trust fundamentally flawed models. Proponents of this argument instead champion *trustworthiness*. Advocates of trustworthiness are of the opinion that many aspects of clinical medicine (and of clinical research)—such as laboratory assays, the complete mechanisms of certain medications, and statistical tests—that are not well or widely understood continue to be used because they have been shown to work reliably and well, even if *how* or *why* remains opaque to many end users [80]. This philosophical barrier has more recently become an operational barrier as well with the passage of the European Union’s General Data Protection Regulation, which requires that automated decision-making algorithms provide “meaningful information about the logic involved.”

Part of the focus on explainability and trustworthiness is due to a desire to understand whether ML algorithms are introducing *bias* into model output, as was notably shown to be the case in a highly publicized series of ProPublica articles about recidivism prediction algorithms [81]. Bias in clinical research–focused algorithms has the potential to be equally devastating, for instance, by theoretically suggesting non-representative study cohorts on the basis of a lower predicted participant drop-out.

### Guidelines toward overcoming operational and philosophical barriers to ML in clinical research

Because the operational problems previously detailed can potentiate the philosophical tangles of ML use in clinical research, many of the ways to overcome these hurdles overlap. The first and foremost approach to many of these issues includes data provenance, quality, and access. The open-access data sources previously discussed (MIMIC, UK Biobank) are good places to start, but inadequate on their own. Enhanced access to data and the technical expertise required to analyze it is needed. Attempts to render health data interoperable have been ongoing for decades, yielding data standard development initiatives and systems, such as the PCORnet Common Data Model [82], FHIR [83], i2b2 [84], and OMOP [85]. Recently, regulation requiring health data interoperability through use of core data classes and elements has been enacted by the US Department of Health and Human Services and Centers for Medicare and Medicaid Services on the basis of the 21st Century Cures Act [85, 86]. Where barriers to data sharing persist, other options to improve the amount of data available include federated data and cloud-based data access, in which developers can train and validate models on data that they do not own or directly interact with [87–89]. This has become increasingly common in certain fields, such as genomics and informatics, as evidenced by large consortia, such as eMERGE and OHDSI [90, 91].

Recently, a group of European universities and pharmaceutical companies have joined to create “MELODDY,” in which large amounts of drug development data will be shared while protecting companies’ proprietary information, though no academic publications have yet been produced [91]. “Challenges” in which teams compete to accomplish ML tasks often yield useful models, such as early sepsis prediction or more complete characterization of breast cancer cell lines, which can then be distributed to participating health institutions for validation in their local datasets [92–95].

Algorithm validation can both help ensure that ML models are appropriate for their intended clinical research use while also increasing stakeholder confidence in the use of ML in clinical research. Though the specifics continue to be debated, published best practices for specific use cases are emerging [96]; recent suggestions to standardize such reporting in a one-page “model card” are notable [97]. For instance, possible model characteristics that could be reported include the intended use cohort, intended outcome of interest, required input data structure and necessary transformations, model type and structure, training cohort specifics, consequences of model application outside of intended use, and algorithm management of uncertainty. Performance metrics that are useful for algorithm evaluation in clinical contexts include receiver-operating characteristic and precision-recall curves, calibration, net benefit, and c-statistic for benefit [92]. Depending on the intended use case, the most appropriate metrics to report or to optimize will differ. For instance, a model intended to identify patients at high risk for protocol non-adherence may have a higher tolerance for false-positives than one intended to simulate study drug dosages for trial planning. Consensus decisions about obligatory metrics for certain model structures and use cases are required to ensure that models with similar intended uses can be compared with one another. Developers will need to specify how often these metrics should be re-evaluated to assess for model drift. Ideally, evaluation of high-stakes clinical research models should be overseen by a neutral third party, such as a regulatory agency.

To foster trustworthiness even in the absence of explainability, it is essential that the model development and validation processes be *transparent*, including the reporting of model uncertainty. This may allow more advanced consumers to evaluate the model from a technical standpoint while at the very least helping less-advanced users to identify situations in which a model’s output should be approached with caution. For instance, understanding the source, structure, and drawbacks of the data used for model training and validation will provide insight into how the model’s output might be affected by the quality of the underlying data. However, trustworthiness may be built by running ML models in clinical research contexts in parallel with traditional research methods to show that the ML methods perform at least as well as traditional approaches. Though the importance of these principles may appear self-evident, the large number of ML models being used commercially for clinical research without reporting of the models’ development and performance characteristics suggests more work is needed to align stakeholders in this regard. Even while writing this manuscript, in which peer-reviewed publications were used whenever available, we encountered many cases in which the only “evidence” supporting a model’s performance was a commercial entity’s promotional material. In several other instances, the peer-reviewed articles available to support a commercial model’s performance offered no information at all about the model’s development or validation, which, as discussed earlier, is crucial to engendering trustworthiness. Another concerning aspect of commercial ML-enabled clinical research solutions is private companies’ and health care systems’ practice of training, validating, and applying models using patient data under the guise of quality improvement initiatives, thereby avoiding the need for ethical/institutional review board approval or patient consent [93]. This practice puts the entire field of ML development at risk of generating biased models and/or losing stakeholder buy-in (as occurred in dramatic fashion with the UK’s “Care.data” initiative) [94] and illustrates the need to build a more reasonable path toward ethical data sharing and more stringent processes surrounding model development and validation.

**Fig. 3:**
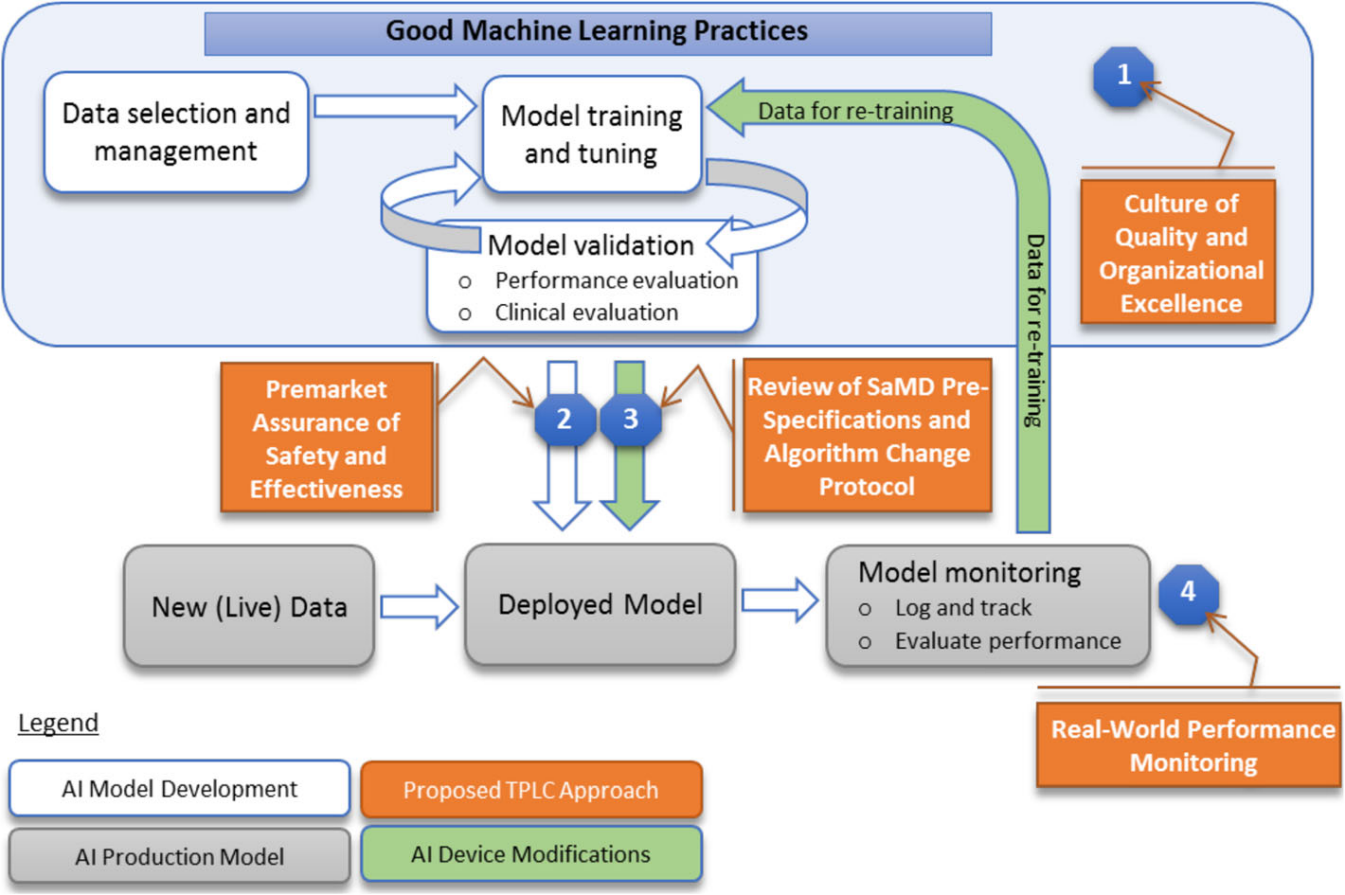
FDA-proposed workflow to regulate machine learning algorithms under the Software as a Medical Device framework. From: Proposed regulatory framework for modifications to artificial intelligence/machine learning (AI/ML)-based software as a medical device: Discussion paper and request for feedback. https://www.fda.gov/media/122535/download. Accessed 17 May 2020

Although no FDA guidance is yet available specific to ML in clinical research, guidance on ML in clinical care and commentary from FDA representatives suggest several possible features of a regulatory approach to ML in clinical research. For instance, the FDA’s proposed ML-specific modifications to the “Software as a Medical Device” Regulations (SaMD) draw a distinction between fixed algorithms that were trained using ML techniques but frozen prior to deployment and those that continue to learn “in the wild.” These latter algorithms may more powerfully take advantage of the large amounts of data afforded by ongoing use but also pose additional risks of model drift with the potential need for iterative updates to the algorithm. In particular, model drift should often be *expected* because models that are incorporated into the decision-making process will inherently change the data they are exposed to in the future. The proposed ML-specific modifications to SaMD guidance outline an institution or organization-level approval pathway that would facilitate these ongoing algorithm updates within pre-approved boundaries (Fig. 3).

The optimal frequency of model re-evaluation by the FDA has yet to be determined (and may vary based off the model type, training set, and intended use), but clearly some form of recurrent review will be needed, prompted either by a certain time period, certain events (for instance, a global pandemic), or both. Discussion with representatives from the FDA indicates that ML in clinical research is viewed as a potentially high-risk use case due to the potential to propagate errors or biases through the algorithm into research studies; however, its potential opportunities were widely appreciated. Until formalized guidance about ML in clinical research is released, the FDA has clearly stated a willingness to work with sponsors and stakeholders on a case-by-case basis to determine the appropriate role of ML in research intended to support a regulatory application. However, this regulatory uncertainty could potentially stifle sponsors’ and stakeholders’ willingness to invest in ML for clinical research until guidance is drafted. This, in turn, may require additional work at a legislative level to provide a framework for further FDA guidance.

Concerns of bias are central to clinical research even when ML is not involved: clinical research and care have long histories of gender, racial, and socioeconomic bias [95, 96]. The ability of ML to potentiate and perpetuate bias in clinical research, possibly without study teams’ awareness, must be actively managed. To the extent that bias can be identified, it can often be addressed and reduced; a worst-case scenario is application of a model with unknown bias in a new cohort with high-stakes results. As with much of ML in clinical research, data quality and quantity are critical in combating bias. No single perfect dataset exists, especially as models trained on real-world data will replicate the intentional or unintentional biases of the clinicians and researchers who generated those data [97]. Therefore, training models on more independent and diverse datasets decreases the likelihood of occult bias [98]. Additionally, bias reduction can be approached through the model construction itself, such as by de-biasing word embeddings and using counterfactual fairness [99–102]. Clinical research teams may pre-specify certain subgroups of interest in which the algorithm must perform equally well [103]. Finally, while ML raises the specter of reinforcing and more efficiently operationalizing historical discrimination, ML may help us de-bias clinical research and care by monitoring and drawing attention to bias [98]. Bias reduction is an area of ML in clinical research in which multi-disciplinary collaboration is especially vital and powerful: clinical scientists may be able to share perspective on long-standing biases in their domains of expertise, while more diverse teams may offer innovative insights into de-biasing ML models.

## Conclusion

While traditional double-blinded, randomized, controlled clinical trials with their associated statistical methodologies remain the gold standard for biomedical evidence generation, augmentation with ML techniques offers the potential to improve the success and efficiency of clinical research, increasing its positive impact for all stakeholders. To the extent that ML-enabled clinical research can improve the efficiency and quality of biomedical evidence, it may save human lives and reduce human suffering, introducing an ethical imperative to explore this possibility. Realizing this potential will require overcoming issues with data structure and access, definitions of outcomes, transparency of development and validation processes, objectivity of certification, and the possibility of bias. The potential applications of ML to clinical research currently outstrip its actual use, both because few prospective studies are available about the relative effectiveness of ML versus traditional approaches and because change requires time, energy, and cooperation. Stakeholder willingness to integrate ML into clinical research relies in part on robust responses to issues of data provenance, bias, and validation as well as confidence in the regulatory structure surrounding ML in clinical research. The use of ML algorithms whose development has been opaque and without peer-reviewed publication must be addressed. The attendees of the January 2020 conference on ML in clinical research represent a broad swath of stakeholders with differing priorities and clinical research–related challenges, but all in attendance agreed that communication and collaboration are essential to implementation of this promising technology. Transparent discussion about the potential benefits and drawbacks of ML for clinical research and the sharing of best practices must continue not only in the academic community but in the lay press and government as well to ensure that ML in clinical research is applied in a fair, ethical, and open manner that is acceptable to all.

## Data Availability

Not applicable

## Abbreviations

EHR: Electronic health record
FDA: US Food and Drug Administration
ML: Machine learning
NLP: Natural language processing
SaMD: Software as a Medical Device
