# Correction to: The role of machine learning in clinical research: transforming the future of evidence generation

**DOI:** 10.1186/s13063-021-05571-4

**Published:** 2021-09-06

**Authors:** E. Hope Weissler, Tristan Naumann, Tomas Andersson, Rajesh Ranganath, Olivier Elemento, Yuan Luo, Daniel F. Freitag, James Benoit, Michael C. Hughes, Faisal Khan, Paul Slater, Khader Shameer, Matthew Roe, Emmette Hutchison, Scott H. Kollins, Uli Broedl, Zhaoling Meng, Jennifer L. Wong, Lesley Curtis, Erich Huang, Marzyeh Ghassemi

**Affiliations:** 1 Duke Clinical Research Institute, Duke University School of Medicine, Box 2834, Durham, NC 27701 USA; 2 Microsoft Research, Cambridge, MA USA; 3 AstraZeneca, Gothenburg, Sweden; 4 Courant Institute of Mathematical Science, New York University, New York, NY USA; 5 Englander Institute for Precision Medicine, Weill Cornell Medical College, New York, NY USA; 6 Northwestern University Clinical and Translational Sciences Institute, Northwestern University, Chicago, IL USA; 7 Division Pharmaceuticals, Open Innovation and Digital Technologies, Bayer AG, Wuppertal, Germany; 8 University of Alberta, Edmonton, Alberta Canada; 9 Department of Computer Science, Tufts University, Medford, MA USA; 10 Billion Minds, Inc., Seattle, WA USA; 11 Verana Health, San Francisco, CA USA; 12 Boehringer-Ingelheim, Burlington, Canada; 13 Sanofi, Cambridge, MA USA; 14 Sanofi, Washington, DC USA; 15 Duke Forge, Durham, NC USA; 16 Vector Institute, University of Toronto, Toronto, Ontario Canada; 17 Department of Electrical Engineering and Computer Science, Massachusetts Institute of Technology, Cambridge, Massachusetts 02139 USA; 18 Institute for Medical Engineering and Science, Massachusetts Institute of Technology, Cambridge, Massachusetts 02139 USA; 19 CIFAR AI Chair, Vector Institute, Toronto, Ontario Canada

**Correction to: Trials 22, 537 (2021)**

**https://doi.org/10.1186/s13063-021-05489-x**

Following the publication of the original article [1], we were notified that current affiliations 17, 18 and 19 were erroneously added to the first author rather than the senior author (Marzyeh Ghassemi).

The original article has now been corrected.
